# Understanding the clinical and demographic characteristics of second coronavirus spike in 192 patients in Tehran, Iran: A retrospective study

**DOI:** 10.1371/journal.pone.0246314

**Published:** 2021-03-19

**Authors:** Hamid Zaferani Arani, Giti Dehghan Manshadi, Hesam Adin Atashi, Aida Rezaei Nejad, Seyyed Mojtaba Ghorani, Soheila Abolghasemi, Maryam Bahrani, Homayoon Khaledian, Pantea Bozorg Savodji, Mohammad Hoseinian, Atefe Kazemzade Bejandi, Shahla Abolghasemi

**Affiliations:** 1 Young Researchers and Elite Club, Tehran Medical Sciences, Islamic Azad University, Tehran, Iran; 2 Department of Anesthesiology and Critical Care, Tehran Medical Sciences, Islamic Azad University, Tehran, Iran; 3 Stem Cell and Regenerative Medicine Center of Excellence, Tehran University of Medical Sciences, Tehran, Iran; 4 Department of Infectious Diseases, Tehran Medical Sciences, Islamic Azad University, Tehran, Iran; 5 Department of Emergency Medicine, Faculty of Medicine, Tehran Medical Sciences, Islamic Azad University, Tehran, Iran; 6 Tehran Medical Sciences, Islamic Azad University, Tehran, Iran; Azienda Ospedaliero Universitaria Careggi, ITALY

## Abstract

During the last months of the coronavirus pandemic, with all those public restrictions and health interventions, the transmission of severe acute respiratory syndrome coronavirus 2 (SARS-CoV-2) appears now to have been raised in some countries around the world. Iran was one of those first countries facing the second wave of coronavirus, due to the lack of appropriate public restrictions because of economic problems the country is facing. The clinical and demographic characteristics of severe cases and non-severe cases of Coronavirus Disease (COVID-19) in 192 patients in Tehran, Iran, between June 16 and July 11, 2020, were investigated. The patients were divided into severe cases (n = 82) and non-severe cases (n = 110). Demographic and clinical characteristics were compared between the two study clusters. The mean age was 54.6 ± 17.2 years, and the most common presenting symptom was persistent cough (81.8%) and fever (79.7%). The logistic regression model revealed that age, BMI, and affected family members were statistically associated with severity. Patients with complicated conditions of disorders faced more hospitalization days and medical care than the average statistical data. As the coronavirus spike in the case and death reports from June 2020, we observed the rise in the incidence of severe cases, where 42.7% (82/192) of cases have resulted in severe conditions. Our findings also suggested that the effect of IFB (Betamethasone) was more valid than the other alternative drugs such as LPV/r and IVIg.

## Introduction

The sudden and unusual outbreak of coronavirus pandemic, which began in Wuhan, China, by late 2019 forced World Health Organization (WHO) to organize a Health Emergency of International Concern (PHEIC) in order determine public health risk and raise the immediate international actions against the coronavirus pandemic [1, 2]. Recent daily reports from the Ministry of Health and Medical Education of Iran had demonstrated that several provinces in Iran are now experiencing a continues to rise in the case and death report, indicating the second wave of the COVID-19 epidemic, as of July 30, 2020, the number Iranian infected by coronavirus reached over 300,000 [3]. With all those public restrictions and health interventions, the transmission of severe acute respiratory syndrome coronavirus 2 (SARS-CoV-2) appears now to have been raised in some countries around the world [4]. Iran was one of those first countries facing the second wave of coronavirus, due to the lack of appropriate public restrictions because of economic problems faced by unfair sanctions against the country [5, 6]. An understanding of how the clinical parameters are acting in this new wave of coronavirus epidemic may help administrators make proper health interventions.

Coronaviruses, causing diseases in animals and humans. Coronaviruses can cause respiratory tract infections, which can result in a mild form (Rhinoviruses), causing common cold, or lethal form (Severe acute respiratory syndrome (SARS-CoV), Middle East respiratory syndrome (MERS-CoV), and COVID-19), which can be severe and even fatal [7]. SARS-CoV (severe acute respiratory syndrome) and MERS-Cov (Middle East respiratory syndrome) were two pathogenic human coronaviruses that happened in recent decades with thousands of case reports [8, 9], but the MERS-Cov did reach high mortality rates of up to 50% in some populations in the region of Middle East [8]. COVID-19, as an RNA virus, was a new type of coronavirus, named severe acute respiratory syndrome coronavirus 2 (SARS-CoV-2), which was first observed in late 2019, so the disease was named COVID-19 [10, 11]. There are similarities between SARS-CoV and SARS-CoV-2 (leading to COVID-19) genome sequence identity between the two viruses is 79.5% [12]. The SARS-CoV-2 infection can lead to severe conditions and even fatal pneumonia SARS-CoV-2 acts very similar to the way SARS-CoV infection was associated with high mortality [13]. There are pieces of evidence for person-to-person transmission of the SARS-CoV-2 in spaces people share and gather (family members living in the same place, dormitories, etc.), hospitals and health care facilities, and public transport systems (bus, subway, etc.) [14, 15].

During the outbreak of coronavirus, the clinical reports, demographic characteristics of patients admitted to hospitals have been evaluated by studies with a short number of samples. Resent meta-analysis study revealed that the male took a larger percentage with 60% (95% CI [0.54, 0.65]) in the gender distribution of patient with COVID‐19, the discharge rate was validated at the rate of 52% (95% CI [0.34,0.70]), and the fatality rate was 5% (95% CI [0.01,0.11]) similar to other study estimation with an overall mortality of 4.3% [16, 17]. A recent study consisting of 1420 patients with mild or moderate COVID-19 indicated that the most common symptoms are headache (70.3%), loss of smell (70.2%), nasal obstruction (67.8%), cough (63.2%), asthenia (63.3%), myalgia (62.5%), rhinorrhea (60.1%), gustatory dysfunction (54.2%) and sore throat (52.9%) [18]. There is not any effective and validated therapy (including vaccine or any antiviral drugs) for COVID-19 until now, but supportive therapies that ease the symptoms and protect multi-organ function may be beneficial. We can decrease the mortality rate and health care service cost by Identifying the integrated symptoms, clinical and demographic characteristics of patients, and identifying the high-risk groups exposed to ease the mortality rate.

This retrospective study on the clinical and demographic characteristics of 192 patients with COVID-19, divided into severe and no-severe clusters, was administrated at Amir Al-Momenin Teaching Hospital and Bu Ali Hospital in Tehran. The patients and their extracted clinical data belonged to the coronavirus spike in cases and death reports in Iran, which was started during June 2020. We aimed to understand the collected clinical and demographical data during this coronavirus spike, to compare the demographic, clinical reports, and laboratory results of severe and non-severe patients.

## Material and methods

### Study design

This retrospective cohort study was conducted based on the result obtained from 192 patients with confirmed COVID-19, hospitalized at Amir Al-Momenin Teaching Hospital and Bu Ali Hospital from June 16 to July 11, 2020, in Tehran, Iran. During this period, another increase in the number of infected patients was observed in Iran, indicating the second wave of COVID-19 pandemic, which was observed in some populated parts of Iran, including Tehran province and the capital city of Tehran. The final date of follow-up was July 18, 2020.

### Patients

All 192 patients’ medical reports analyzed by this study, were selected from visiting patients with positive COVID-19 test, which was done by the specific real-time PCR Kit test named, HBRT-COVID-19 (Chaozhaou Hybribio Biochemistry Ltd, China). The throat swab samples collected from visiting patients were collected and SARS-CoV-2 disease detection was done by molecular recognition through real-time PCR (polymerase chain reaction) Assay Testing using real-time reverse transcription-polymerase chain reaction for diagnostic purposes.

### Clinical data collection

In this study, we analyzed the medical records of patients registered at the two-mentioned hospital in the city of Tehran. Our researchers collected clinical information, such as the medical history, demographic data (gender, and age), epidemiology, exposure history (be in contact with infected, suspects patients or even traveled), signs and symptoms (such as fever, cough, shortness or difficulties in the act of breathing, fatigue, anorexia, hemoptysis, sputum production, dyspnea, Myalgia, Pharyngalgia, nausea, vomiting, Diarrhoea, Headache, Abdominal pain, Dizziness, etc.), and routine blood laboratory tests (including blood type, white blood cell count, Rhesus (Rh) factor), which were obtained by the hospitals’ staff. We did search the patient medical history for any underlying diseases, treatment, and prognosis. These diseases include Gastrointestinal disorder, Chronic obstructive pulmonary disease (COPD), cardiovascular disease (CVD), hypercholesterolemia (HCL), hypertension (HTN), myocardial infarction (MI), hematologic diseases, endocrine disease, neurological diseases, rheumatism, kidney failure and any type of cancer. Smoking history and drug usage of the patients were recorded as provided. The medicine usage of patients was recorded (medicines such as Hydroxychloroquine, Azithromycin). Our team of related physicians did review the original documents and the extracted data several times. The clinical data collection based on database assessment and their analysis was conducted from 12^th^ July to 19^th^ July.

**Fig 1:**
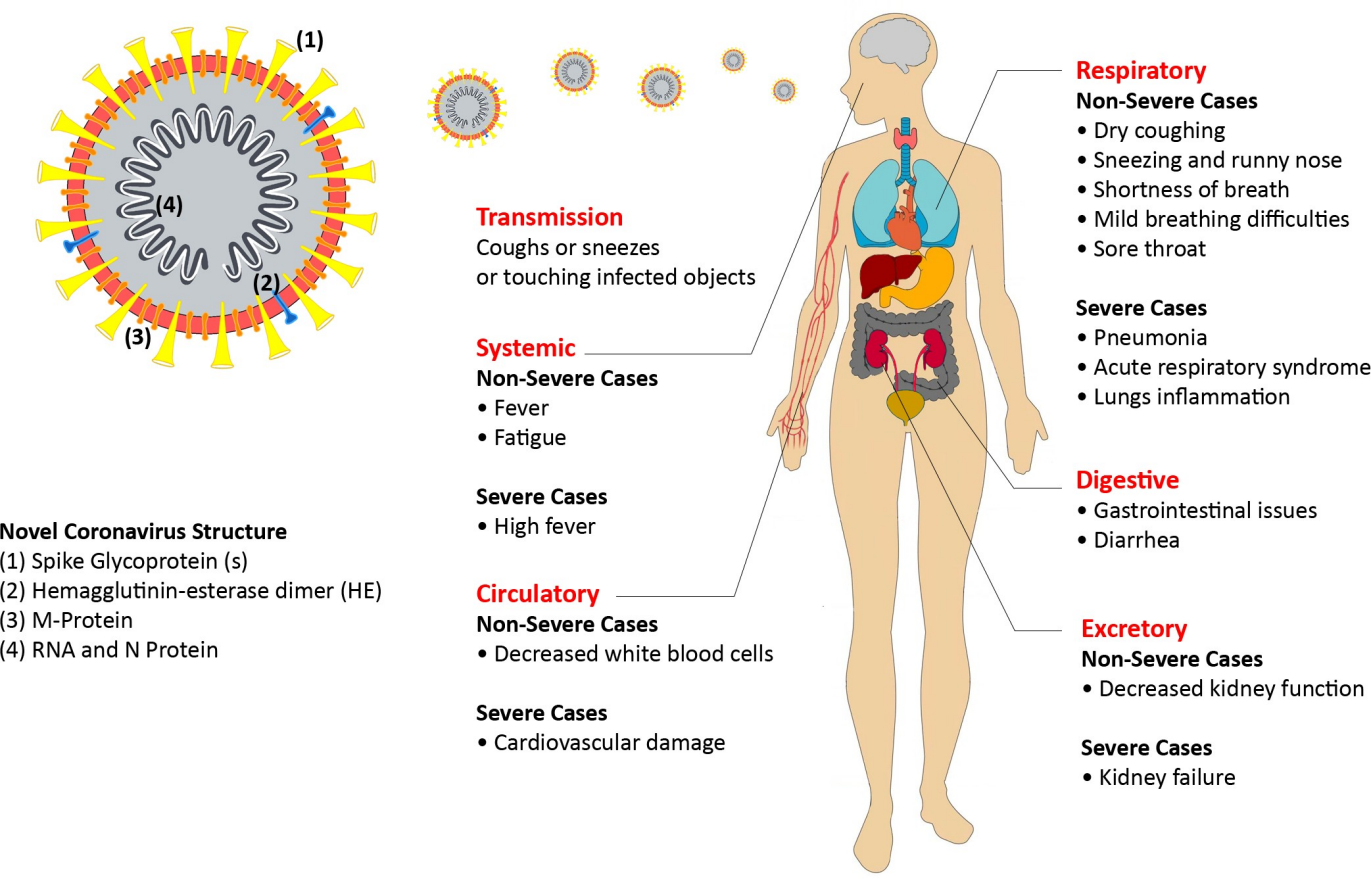
COVID-19 signs and symptoms, and the structure of Novel Coronavirus 2019.

In this study, the 192 coronaviruses conformed patients were divided into two clusters of severe and non-severe patients. There was some patients with non-severe symptoms at first days of administration at hospitals, but by developing the situation, they were categorized in a severe cluster. The severe cases consist of any patients with these criteria: a) any organ failure requiring ICU monitoring, b) if the rate of breathing was more than 30 times/min (indicating the respiratory distress), c) if the oxygen saturation level was less than 93%, d) observing any need for mechanical ventilation in excuse of respiratory failure and Ling inflammation. The sign and symptoms of COVID-19 had been demonstrated in Fig 1.

### Statistical analysis

Descriptive analyses of the variables were expressed as number (percentages) or mean ± SD (Standard Deviation), or median, Q_1_, and Q_2_, where interquartile range (IQR) is equal to Q_1_—Q_3_. Mean ± standard deviation and number (percentage) were used to present values of the quantitative and qualitative variables, respectively. Data were assessed for the normality assumption by the Kolmogorov-Smirnov test. To compare the study variables among the severe and non-severe clusters, Pearson Chi-Square or Fisher’s exact tests for qualitative variables and Independent T, Mann-Whitney U or Kruskal-Wallis tests for quantitative variables were applied. Finally, a multiple logistic regression model using the stepwise method was conducted to evaluate the association of severity with study variables. All statistical analyzes were performed Using SPSS version 24 (IBM, USA) and *P*-values less than 0.05 were considered significant statistically.

### Ethics approval

The Ethics Committee of Islamic Azad University, Tehran Medical Sciences University (IAUTMS), approved this study. Due to the nature of this study, the committee waived the requirement for informed consent, by signing an agreement on keeping participants’ information secret.

## Results

This study included 192 coronavirus infected patients, with a mean age of 54.6 ± 17.2 years old (48.2 ± 16.3 was the average age of the non-severe cluster, while the severe cluster was older by 63.3 ± 15.1), most of the patients were overweight (as BMI indicated), and the majority belonged to male gender with 102 patients (53.7%), accompanied by 88 (46.3%) female patients. The average duration of hospitalization was about 8 days with a standard deviation of 5 days. The cases were clinically divided into severe and non-severe clusters. As the clinical and demographic data gathered in Table 1 indicates, patients of the severe cluster were more involved with underlying diseases (35.1% of severe patients did not have any underlying disease, but 51.2% of non-severe patients experienced the same). Information on vital signs and symptoms at the first visit was also presented in Table 1. The most commonly observed symptoms of COVID-19 were cough (81.8%), fever (79.7%), and headache (70.3%). Severe and non-severe patients were statistically different in terms of these symptoms. Fever was more involved with non-severe patients by 96.3% against 67.3%, and cough mostly experienced by non-severe patients, by 95.1% against 71.8%. Being Fatigue, anorexia, dyspnea, chest tightness, myalgia, nausea, vomiting, diarrhea, and abdominal pain, also involved more with non-severe patients. There were a few dizziness experiences only with some patients who belonged to the non-severe cluster.

**Table 1:** Clinical and demographic characteristics for 192 cases involved in this study (from which, 82 cases were categorized in the severe group and 110 cases in the non-severe group).

	Total	Non-Severe	Severe	*P*-value
Age (mean ± SD) (years)	54.6 ± 17.2	48.2 ± 16.3	63.3 ± 15.1	<0.001
BMI (mean ± SD)	27.90 ± 4.32	27.38 ± 4.82	28.45 ± 3.67	0.165
BMI				
Normal				0.079
Male	18 (56.3%)	12 (54.5%)	6 (60.6%)	
Female	14 (43.8%)	10 (45.5%)	4 (40.0%)	
Overweight				
Male	32 (53.3%)	14 (53.8%)	18 (52.9%)	
Female	28 (46.7%)	12 (46.2%)	16 (47.1%)	
Obese				
Male	14 (41.2%)	8 (50.0%)	6 (33.3%)	
Female	20 (58.8%)	8 (50.0%)	12 (66.7%)	
Gender				
Male	102 (53.7%)	62 (57.4%)	40 (48.8%)	0.238
Female	88 (46.3%)	46 (42.6%)	42 (51.2%)	
Underlying disease				
Non	84 (51.2%)	58 (64.4%)	26 (35.1%)	<0.001
Internal diseases	48 (29.3%)	26 (28.9%)	22 (29.7%)	
Coronary heart disease	24 (14.6%)	2 (2.2%)	22 (29.7%)	
Neurological disorders	8 (4.9%)	4 (4.4%)	4 (5.4%)	
Number of family members involved in the case	1 ± 1	2 ± 1	1 ± 1	<0.001
Isolation situation of the house	4 ± 1	4 ± 1	5 ± 1	0.740
Time from symptoms to admission	9 ± 5	6 ± 5	10 ± 5	0.001
Time from admission to release	8 ± 5	5 ± 4	9 ± 5	0.001
Having a job				
Yes	62 (47.0%)	40 (57.1%)	22 (35.5%)	0.013
No	70 (53.0%)	30 (42.9%)	40 (64.5%)	
Smoking				
Yes	20 (13.5%)	14 (17.5%)	6 (8.8%)	0.124
No	128 (86.5%)	66 (82.5%)	62 (91.2%)	
Drug usage				
Yes	12 (7.7%)	4 (4.7%)	8 (11.4%)	0.114
No	144 (92.3%)	82 (95.3%)	62 (88.6%)	
Staff member of Hospital				
Yes	4 (2.6%)	4 (4.9%)	0 (0.0%)	0.125
No	148 (97.4%)	78 (95.1%)	70 (100.0%)	
Travel history in the last 14 days				
Yes	6 (4.0%)	4 (4.9%)	2 (2.9%)	0.690
No	144 (96.0%)	78 (95.1%)	66 (97.1%)	
Having contact with a person who has been diagnosed with COVID-19				
Yes	68 (57.6%)	44 (71.0%)	24 (42.9%)	0.002
No	50 (42.4%)	18 (29.0%)	32 (57.1%)	
Having contact with a person who has been suspected with COVID-19				
Yes	78 (58.2%)	32 (47.1%)	20 (30.3%)	0.008
No	56 (41.8%)	36 (52.9%)	24 (42.9%)	
Having contact with animals				
Yes	6 (3.9%)	4 (4.8%)	2 (2.9%)	0.689
No	148 (96.1%)	80 (95.2%)	68 (97.1%)	
Consuming Hydroxychloroquine				
Yes	148 (89.2%)	86 (93.5%)	62 (83.8%)	0.046
No	18 (10.8%)	6 (6.5%)	12 (16.2%)	
Consuming Azithromycin				
Yes	116 (70.7%)	64 (71.1%)	52 (70.3%)	0.906
No	48 (29.3%)	26 (28.9%)	22 (29.7%)	
Rhesus (Rh) factor				
Positive	78 (40.6%)	22 (20.0%)	56 (68.3%)	<0.001
Negative	114 (59.4%)	88 (80.0%)	26 (31.7%)	
Blood Type				
A (positive or negative)	50 (26.0%)	12 (10.9%)	38 (46.3%)	<0.001
B (positive or negative)	50 (26.0%)	46 (41.8%)	4 (4.9%)	
AB (positive or negative)	48 (25.0%)	10 (9.1%)	38 (46.3%)	
O (positive or negative)	44 (22.9%)	42 (38.2%)	2 (2.4%)	
Symptoms				
Fever	153 (79.7%)	79 (96.3%)	74 (67.3%)	<0.001
Cough	157 (81.8%)	78 (95.1%)	79 (71.8%)	<0.001
Fatigue	132 (68.8%)	77 (93.9%)	55 (50.0%)	<0.001
Anorexia	52 (27.1%)	42 (51.2%)	10 (9.1%)	<0.001
Hemoptysis	0 (0.0%)	0 (0.0%)	0 (0.0%)	NA
Sputum production	31 (16.1%)	18 (21.9%)	13 (11.8%)	0.074
Dyspnea	76 (39.6%)	72 (87.8%)	4 (3.6%)	<0.001
Chest tightness	47 (24.5%)	34 (41.5%)	13 (11.8%)	<0.001
Mylagia	59 (30.7%)	53 (64.6%)	6 (5.4%)	<0.001
Pharyngalgia	18 (9.4%)	7 (8.5%)	11 (10.0%)	0.806
Nausea	35 (18.2%)	29 (35.4%)	6 (5.4%)	<0.001
Vomiting	21 (10.9%)	17 (20.7%)	4 (3.6%)	<0.001
Diarrhoea	83 (43.2%)	62 (75.6%)	21 (19.1%)	<0.001
Headache	135 (70.3%)	61 (74.4%)	74 (67.3%)	0.339
Abdominal pain	65 (33.8%)	48 (58.5%)	17 (15.4%)	<0.001
Dizziness	2 (1.0%)	2 (2.4%)	0 (0.0%)	0.181

All of these data had been extracted from their medical document. The common symptoms of COVID-19 had been categorized in this table.

BMI stands for Body Mass Index.

In this study, we did also gather data of vital signs monitored on the admission day such as Maximum number of blood pressure, Minimum number of blood pressure, respiration rate (Rate of breathing per minute), pulse rate, and also percutaneous rate. According to Table 2, about 97% of non-severe patients experienced a lower rate of respiration rate, while 60% of severe clusters experience a rate between 24 and 29 (breath per minute). The pulse rate of patients in non-severe clusters was mostly in the range of 66 to 99 pulse per minute, while about 33% of severe patients experienced higher rates than 100 pulse per minute. The percutaneous oxygen saturation was higher with non-severe patients (94%), but severe patients experience lower levels of percutaneous oxygen saturation (42% lower than 980, and 32% experienced the range of 90 to 93). For patients in the severe cluster, the maximum number of blood pressure and respiratory rate were higher, and even the oxygen saturation was lower within them.

**Table 2:** Vital signs are monitored on the admission day at the hospitals.

	Total	Severe	Non-severe	*P*-value
Maximum number of blood pressure (Systolic)				
<100	18 (9%)	11 (13)	7 (7)	0.108
100–119	47 (25%)	23 (28)	24 (21)	
120–139	104 (54%)	42 (52)	62 (56)	
≥140	23 (12%)	6 (7)	17 (16)	
Minimum number of blood pressure (Diastolic)				
<70	16 (8)	9 (11)	7 (6)	0.003
70–79	45 (24)	25 (31)	20 (18)	
80–89	119 (62)	39 (47)	80 (74)	
≥90	12 (6)	9 (11)	3 (2)	
Respiration rate (Rate of breathing per minute)				
<24	132 (69)	25 (30)	107 (97)	<0.001
24–29	51 (27)	49 (60)	2 (2)	
≥30	9 (4)	8 (10)	1 (1)	
Pulse rate (per minute)				
<60	7 (4)	3 (3)	4 (3)	<0.001
60–99	149 (78)	52 (64)	97 (89)	
≥100	36 (18)	27 (33)	9 (8)	
Percutaneous oxygen saturation				
<90	37 (20)	35 (42)	2 (2)	<0.001
90–93	31 (16)	26 (32)	5 (4)	
≥94	124 (64)	21 (26)	103 (94)	

**Fig 2:**
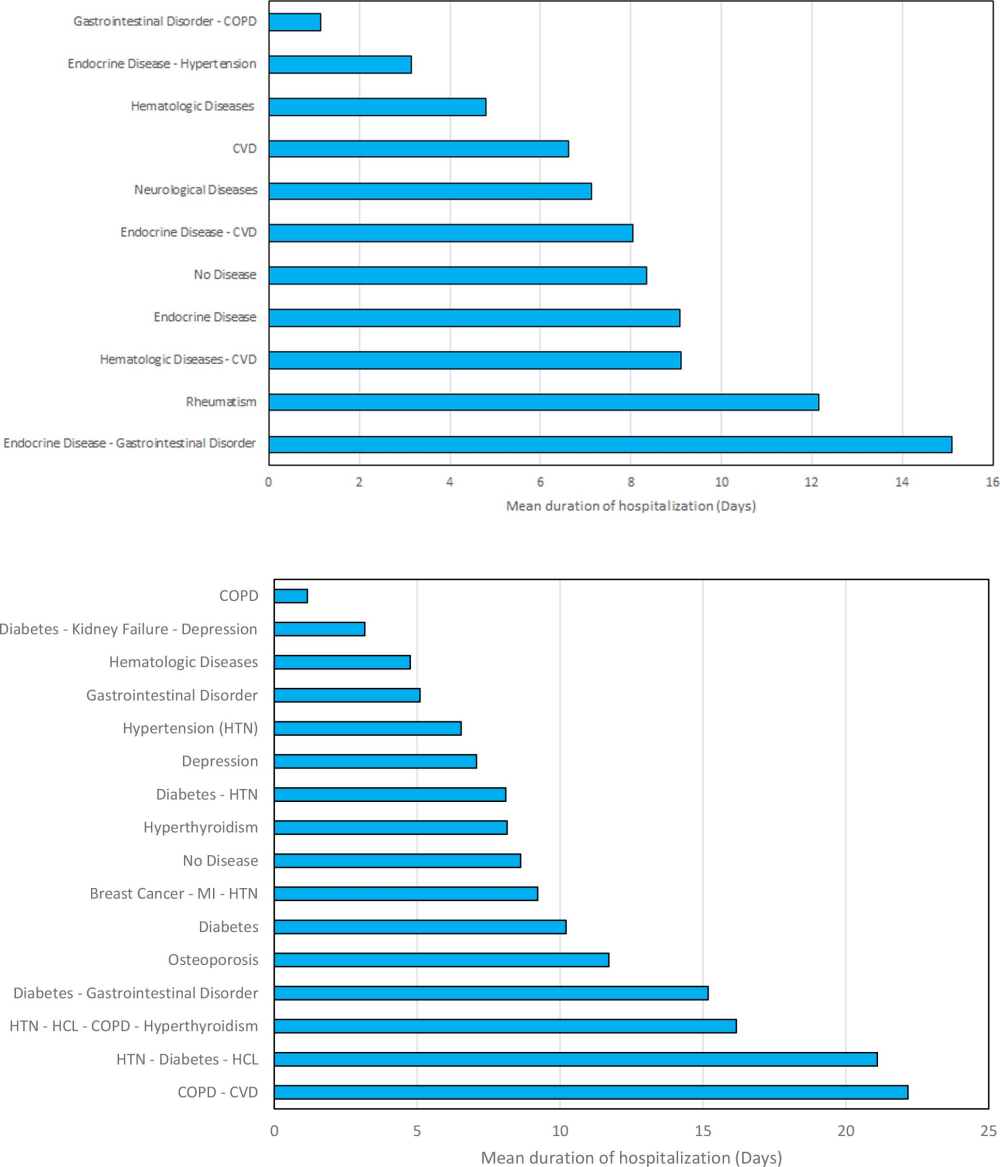
The relationship between patient’s diseases and their average duration of hospitalization for the studied cases. a) for most common disorders involved with patients, b) for more complicated mixtures of diseases, were observed in cases (COPD stands for chronic obstructive pulmonary disease, CVD stands for cardiovascular disease, HCL stands for Hypercholesterolemia, HTN stands for Hypertension, and MI stands for Myocardial Infarction).

Meantime of hospitalization was associated with gender (males countering the longer days of hospitalization), age (the older patients, the more hospitalization days), medication the patients were through, oxygen saturation, blood group, and Rhesus (Rh) factor. Male patients as well as older patients had a higher mean time of hospitalization. The average hospitalization days for males was 3 days more than the average belonged to females. Those patients, who received IVG or those with lower levels of oxygen saturation, also had a higher mean time of hospitalization. Finally, those patients with AB blood type and those with positive Rhesus (Rh) factor experienced more days in the hospital (Table 3). The relationship between the patient’s diseases and the average period they should be under the monitor and treatment of hospital had been demonstrated in Fig 2. Fig 2A suggested patients with both endocrine disease and gastrointestinal disorders were facing more average time of hospitalization (15.09 days), meanwhile, patients without any diseases were facing an average of 8.36 days of hospitalization. Patients with the endocrine disease as the only background disease were faced with a slight increase in the hospitalization time (with an average time of 9.08 days). The impact of more complicated conditions was presented in Fig 2B, suggesting that patients with both COPD and CVD, had to spend more time under medical monitoring (average time of 22.17 days). Patients with complicated conditions of disorders faced more hospitalization days than the average statistical data.

**Table 3:** Relationship between hospitalization time and study variables.

	Mean ± SD	Median	Q_1_	Q_3_	*P*-value
Gender					
Male	10 ± 5	7	6	14	0.045
Female	7 ± 5	7	1	10	
Age (Years)					
< 45	6 ± 5	7	1	10	0.007
45–64	7 ± 4	7	6	10	
≥ 64	10 ± 5	9	6	14	
BMI					
Normal	11 ± 5	13	7	14	0.244
Overweight	9 ± 6	8	5	13	
Obese	8 ± 6	6	1	11	
Medication					
IFB (Betamethasone)	8 ± 8	5	1	16	0.025
Kaletra or Lopinavir/ritonavir (LPV/r)	7 ± 1	7	7	8	
IVIg (Intravenous immunoglobulin)	9 ± 5	11	7	14	
Oxygen saturation (%)					
< 90	9 ± 5	8	6	12	<0.001
90–93	10 ± 4	9	7	14	
> 93	4 ± 6	1	1	3	
Underlying disease					
Non	8 ± 5	7	5	13	0.536
Internal diseases	9 ± 6	8	6	14	
Coronary heart disease	8 ± 5	9	1	12	
Neurological disorders	5 ± 2	5	3	7	
Blood Type					
A (positive or negative)	9 ± 5	7	6	13	<0.001
B (positive or negative)	3 ± 2	3	1	6	
AB (positive or negative)	10 ± 5	9	7	14	
O (positive or negative)	5 ± 4	5	5	8	
Rhesus (Rh) factor					
Yes	7 ± 4	7	5	10	0.028
No	10 ± 6	9	6	14	
Having a job					
Yes	10 ± 5	11	6	14	0.220
No	9 ± 5	8	5	12	
Smoking					
Yes	12 ± 6	12	7	14	0.058
No	8 ± 5	7	5	12	
Drug usage					
Yes	12 ±6	12	7	14	0.099
No	8 ± 5	8	3	12	
Staff member of Hospital					
Yes	-	-	-	-	-
No	9 ± 6	8	5	13	
Travel history in the last 14 days					
Yes	6 ± 0	6	6	6	0.439
No	9 ± 6	8	5	14	
Having contact with a person who has been diagnosed with COVID-19					
Yes	10 ± 7	10	2	15	0.545
No	9 ± 4	8	6	11	
Having contact with a person who has been suspected with COVID-19					
Yes	8 ± 4	7	6	13	0.891
No	9 ± 6	8	5	12	
Having contact with animals					
Yes	10 ± 10	7	1	12	0.946
No	8 ± 5	8	5	13	
Consuming Hydroxychloroquine					
Yes	8 ± 6	7	6	8	0.252
No	8 ± 6	7	3	12	
Consuming Azithromycin					
Yes	9 ± 5	9	5	14	0.239
No	8 ± 6	7	6	8	

The variables were expressed as mean ± Standard Deviation, Median, Q_1_, and Q_3_, where the interquartile range (IQR) is equal to Q_1_—Q_3_.

BMI stands for Body Mass Index.

It was obvious that cases from the severe group were more likely to have shortness of breath. It was also found that oxygen saturation was associated with the medication received (Table 4). The medications such as IFB (Betamethasone), Kaletra or Lopinavir/Ritonavir (LPV/r), and IVIg (Intravenous immunoglobulin) may have good impacts for severe patients, but the effect if IFN (Betamethasone) was more valid, according to our findings. The logistic regression model revealed that age, BMI, and affected family members were statistically associated with severity. Per one unit increase in age and BMI, the risk of severity would increase by 8 and 21 percent, respectively. Moreover, per one person infected in the family, the risk of severity would decrease by 44 percent (Table 5).

**Table 4:** The relationship between the medications such as IFB (Betamethasone), Kaletra, and IVIg and oxygen saturation had been studied.

	<90	90–93	>93	*P-value*
	Number (%)	Number (%)	Number (%)	
IFB (Betamethasone)	15 (22.4)	2 (13.3)	13 (72.2)	< 0.001
Kaletra or Lopinavir/ritonavir (LPV/r)	25 (37.3)	5 (33.3)	0 (0.0)	< 0.001
IVIg (Intravenous immunoglobulin)	27 (40.3)	8 (53.3)	5 (27.8)	< 0.001

**Table 5:** Logistic registration model; association of severity and study variables.

Variable	B	S.E.	OR (95% Cl)	*P-value*
Age	0.078	0.019	1.081 (1.042–1.121)	< 0.001
BMI	0.198	0.084	1.218 (1.034–1.435)	0.018
Number of affected persons in family	-0.572	0.218	0.564 (0.368–0.865)	0.009
Employment	-0.619	0.512	0.539 (0.198–1.468)	0.227

BMI stands for Body Mass Index.

## Discussion

This study was performed on a medical report administrated in Amir Al-Momenin Teaching Hospital and Bu Ali Hospital from June 16 to July 11, 2020. During this period, there was a significant coronavirus spike in the case and death reports, and we reported the incidence of severe cases by 42.7% (82/192). COVID-19 as an infectious disease has infected a large number of people all around the world and countries such as Iran [19]. According to recent studies around the world, the clinical manifestations of coronavirus patients are not consistent or having a fixed pattern and so the fertility rate and percentage of severe patients are very different [20]. The incidence of severe cases by a recent meta-analysis was the same as our findings, and it may reflect the same complicated wave of the epidemic taking place in Iran [17]. The male gender, with 102 cases (53.7%) were more exposed to the risk of infection with coronavirus, and the available BMI of part of the participated cases indicated that the male gender did have a higher chance of over-weight than females. The factor of age in patients induces the risk of death and fertility mainly because of declined immune system ability [21] and our logistic regression results indicated the correlation between age and fertility risk (OR = 1.08, 95% CI: 1.04–1.12). The average time from the onset of symptoms to admission at the hospital, about 6 ± 5 days in non-severe patients but severe patients experienced a higher average time of 10 ± 5 days for the diagnostician of their coronavirus infection; but in contrast, the non-severe cases spend less hospitalization time (5 ± 4, against 9 ± 5). Patients with complicated conditions of disorders especially do suffering from two different disorders, may face more hospitalization days and medical care than the average statistical data suggested. Our study suggested patients with both COPD and CVD, may have to be under the medical health care of the hospital more than any other patients (average time of 22.17 days).

To date, there was not any recommended and high-effective treatment for COVID-19 patients, but there were some reports on the efficient effect of some anti-inflammatory drugs for severe patients with chronic lung conditions [22]. Most patients undergo treatments dealing with the control of their hydration, nutrition, and fever [23]. In severe cases, those suffering from hypoxia, mechanical ventilation, high flow nasal cannula, and face mask may supply the inhaling oxygen [24, 25]. Some anti-viral drugs can be used as anti- SARS-CoV-2 agents including interferon-alpha (IFNα), Remdesivir, Betamethasone (IFB) Lopinavir/ritonavir (Kaletra), and Arbidol [24–26]. There were patients receiving medicines for their current treatment (any background diseases) or their severe conditions due to coronavirus infection. These medications including IFB (Betamethasone), Kaletra or Lopinavir/Ritonavir (LPV/r), and IVIg (Intravenous immunoglobulin) may have good impacts for severe patients, but there is not any Food and Drug Administration approval on it. Betamethasone is a steroid prevents the release of substances in the body, which causes inflammation [26]. IFB can be prescribed for use in the treatment of many different inflammatory conditions such as allergic reactions, multiple sclerosis, and inflammation of the joints or tendons, and problems caused by low adrenal gland hormone levels [27]. Recent studies indicated that IFNα inhibits the replication of SARS-CoV *in vitro*, suggesting that IFNα can be considered as a potential drug candidate for COVID-19 therapy [26]. Lopinavir/Ritonavir with the brand name of Kaletra can slow down HIV, the infection that causes AIDS [28]. Intravenous Immunoglobulin Therapy (IVIg) can help people with weakened immune systems or other diseases fight off infections such as chronic inflammatory demyelinating polyneuropathy [29]. The findings suggested that the effect of IFB (Betamethasone) was more valid than the others were.

In conclusion, this study aimed to run a retrospective study on the clinical and demographic characteristics of 192 patients with COVID-19, divided into severe and no-severe clusters, in Tehran, the capital city of Iran. This study was conducted during the second coronavirus spike in cases and death reports in Iran. With the appearance of the pandemic, many researchers and clinicians struggle to identify symptoms and characterization of the pandemic and even propose effective drugs dealing with it. We found demographic and clinical differences between severe and non-severe clusters. It can be suggested that patients of male gender and elderly people may face more probably severe conditions, just as other recent studies suggested [18]. The most common symptoms of COVID-19 were cough (81.8%), fever (79.7%), headache (70.3%), and Fatigue (68.8%). The most hospitalization period achieved by patients suffering from COPD and CVD (22.17 days) and HTN, Diabetes, and HCL (21.10 days). Our findings also suggested that the effect of IFB (Betamethasone) was more valid than the other alternative drugs used, such as LPV/r and IVIg. We hope by further studies, the mortality rate and health care service cost may be decreased by identifying the integrated symptoms, clinical and demographic characteristics of patients, suggesting alternative medicines, and identifying the high-risk groups exposed to ease the mortality rate.

## Supporting information

S1 Abstract figure Schematic view of the design and current study process. (TIF)

## References

[1] Chen Y, Liu Q, Guo D. Emerging coronaviruses: genome structure, replication, and pathogenesis. J Med Virol. 2020;92(4):418–23. 10.1002/jmv.25681 31967327 PMC7167049

[2] WHO. Remarks by Dr Michael Ryan, Executive Director, WHO Health Emergencies Programme at media briefing on COVID-19 on 13 February 2020 https://www.who.int/news-room/detail/13-02-2020-remarks-by-dr-michael-ryan-executive-director-who-health-emergencies-programme-at-mediabriefing-on-covid-19-on-13-february-20202020

[3] Education MoHaM. Over 300,000 Iranians Infected by Coronavirus: Health Ministry: http://irangov.ir/detail/344270; 2020

[4] Hou C, Chen J, Zhou Y, Hua L, Yuan J, He S, et al. The effectiveness of quarantine of Wuhan city against the Corona Virus Disease 2019 (COVID‐19): A well‐mixed SEIR model analysis. J Med Virol. 2020. 10.1002/jmv.25827 32243599

[5] Nikpouraghdam M, Farahani AJ, Alishiri G, Heydari S, Ebrahimnia M, Samadinia H, et al. Epidemiological characteristics of coronavirus disease 2019 (COVID-19) patients in IRAN: A single center study. J Clin Virol. 2020. 10.1016/j.jcv.2020.104378 32353762 PMC7172806

[6] Takian A, Raoofi A, Kazempour-Ardebili S. COVID-19 battle during the toughest sanctions against Iran. Lancet (London, England). 2020;395(10229):1035. 10.1016/S0140-6736(20)30668-1 32199073 PMC7138170

[7] Cui J, Li F, Shi Z-L. Origin and evolution of pathogenic coronaviruses. Nat Rev Microbiol. 2019;17(3):181–92. 10.1038/s41579-018-0118-9 30531947 PMC7097006

[8] Andersen KG, Rambaut A, Lipkin WI, Holmes EC, Garry RF. The proximal origin of SARS-CoV-2. Nat Med. 2020;26(4):450–2. 10.1038/s41591-020-0820-9 32284615 PMC7095063

[9] Organization WH. Middle East respiratory syndrome coronavirus (MERS-CoV). 2016.

[10] Huang C, Wang Y, Li X, Ren L, Zhao J, Hu Y, et al. Clinical features of patients infected with 2019 novel coronavirus in Wuhan, China. Lancet. 2020;395(10223):497–506. 10.1016/S0140-6736(20)30183-5 PMC7159299 31986264

[11] Chen N, Zhou M, Dong X, Qu J, Gong F, Han Y, et al. Epidemiological and clinical characteristics of 99 cases of 2019 novel coronavirus pneumonia in Wuhan, China: a descriptive study. Lancet. 2020;395(10223):507–13. 10.1016/S0140-6736(20)30211-7 32007143 PMC7135076

[12] Zhou Y, Hou Y, Shen J, Huang Y, Martin W, Cheng F. Network-based drug repurposing for novel coronavirus 2019-nCoV/SARS-CoV-2. Cell Discov. 2020;6(1):1–18. 10.1038/s41421-020-0153-3 32194980 PMC7073332

[13] Zhou F, Yu T, Du R, Fan G, Liu Y, Liu Z, et al. Clinical course and risk factors for mortality of adult inpatients with COVID-19 in Wuhan, China: a retrospective cohort study. Lancet. 2020. 10.1016/S0140-6736(20)30566-3 32171076 PMC7270627

[14] Li C, Ji F, Wang L, Wang L, Hao J, Dai M, et al. Asymptomatic and human-to-human transmission of SARS-CoV-2 in a 2-family cluster, Xuzhou, China. Emerg Infect Dis. 2020;26(7):1626. 10.3201/eid2607.200718 32228809 PMC7323514

[15] Jiang X-L, Zhang X-L, Zhao X-N, Li C-B, Lei J, Kou Z-Q, et al. Transmission potential of asymptomatic and paucisymptomatic SARS-CoV-2 infections: a three-family cluster study in China. J Infect Dis. 2020. 10.1093/infdis/jiaa206 PMC7188140 32319519

[16] Jordan RE, Adab P, Cheng K. Covid-19: risk factors for severe disease and death. Br Med J. 2020. 10.1136/bmj.m1198 32217618

[17] Lq Li, Huang T Wang Yq, Zp Wang, Liang Y Huang Tb, et al. COVID‐19 patients’ clinical characteristics, discharge rate, and fatality rate of meta‐analysis. J Med Virol. 2020;92(6):577–83. 10.1002/jmv.25757 32162702 PMC7228329

[18] Lechien JR, Chiesa‐Estomba CM, Place S, Van Laethem Y, Cabaraux P, Mat Q, et al. Clinical and epidemiological characteristics of 1,420 European patients with mild‐to‐moderate coronavirus disease 2019. J Inter Med. 2020;288(3):335–344. 10.1111/joim.13089 32352202 PMC7267446

[19] Mo P, Xing Y, Xiao Y, Deng L, Zhao Q, Wang H, et al. Clinical characteristics of refractory COVID-19 pneumonia in Wuhan, China. Clin Infect Dis. 2020. 10.1093/cid/ciaa270 32173725 PMC7184444

[20] Guillen E, Pineiro GJ, Revuelta I, Rodriguez D, Bodro M, Moreno A, et al. Case report of COVID‐19 in a kidney transplant recipient: does immunosuppression alter the clinical presentation? Am J Transplant. 2020. 10.1111/ajt.15874 PMC7228209 32198834

[21] Koff WC, Williams MA. Covid-19 and immunity in aging populations—a new research agenda. N Engl J Med. 2020. 10.1056/NEJMp2006761 32302079

[22] Little P. Non-steroidal anti-inflammatory drugs and covid-19. Br Med J. 2020. 10.1136/bmj.m1185 32220865

[23] Singhal T. A review of coronavirus disease-2019 (COVID-19). Indian J Pediatr. 2020;13:1–6. 10.1007/s12098-020-03263-6 PMC7090728 32166607

[24] Li GQ, Zhao J, Tu ZT, Li JB, Liu QQ, Shi LQ, et al. Treating influenza patients of wind-heat affecting Fei syndrome by jinhua qinggan granule: a double-blinded randomized control trial. Chinese J Integr Med. 2013;33(12):1631–5. 24517059

[25] Li H, Liu SM, Yu XH, Tang SL, Tang CK. Coronavirus disease 2019 (COVID-19): current status and future perspective. International journal of antimicrobial agents. 2020 3 29:105951. 10.1016/j.ijantimicag.2020.105951 32234466 PMC7139247

[26] McIntosh JJ. Corticosteroid guidance for pregnancy during COVID-19 pandemic. Am J Perinatol. 2020;37(8):809. 10.1055/s-0040-1709684 32274772 PMC7356057

[27] Kuypers E, Collins JJ, Kramer BW, Ofman G, Nitsos I, Pillow JJ, et al. Intra-amniotic LPS and antenatal betamethasone: inflammation and maturation in preterm lamb lungs. American Am J Physiol Lung Cell Mol Physiol. 2012;302(4):L380–L9. 10.1152/ajplung.00338.2011 22160306 PMC3289264

[28] Wan S, Xiang Y, Fang W, Zheng Y, Li B, Hu Y, et al. Clinical features and treatment of COVID‐19 patients in northeast Chongqing. J Med Virol. 2020. 10.1002/jmv.25783 32198776 PMC7228368

[29] Shoenfeld Y. Corona (COVID-19) time musings: our involvement in COVID-19 pathogenesis, diagnosis, treatment and vaccine planning. Autoimmunity Reviews. 2020. 10.1016/j.autrev.2020.102538 PMC7131471 32268212

