# Supplementary figures and images for: Understanding the clinical and demographic characteristics of second coronavirus spike in 192 patients in Tehran, Iran: A retrospective study
Source: PLoS One. 2021 Mar 19;16(3):e0246314. doi: 10.1371/journal.pone.0246314 (PMC7979149; doi:10.1371/journal.pone.0246314)

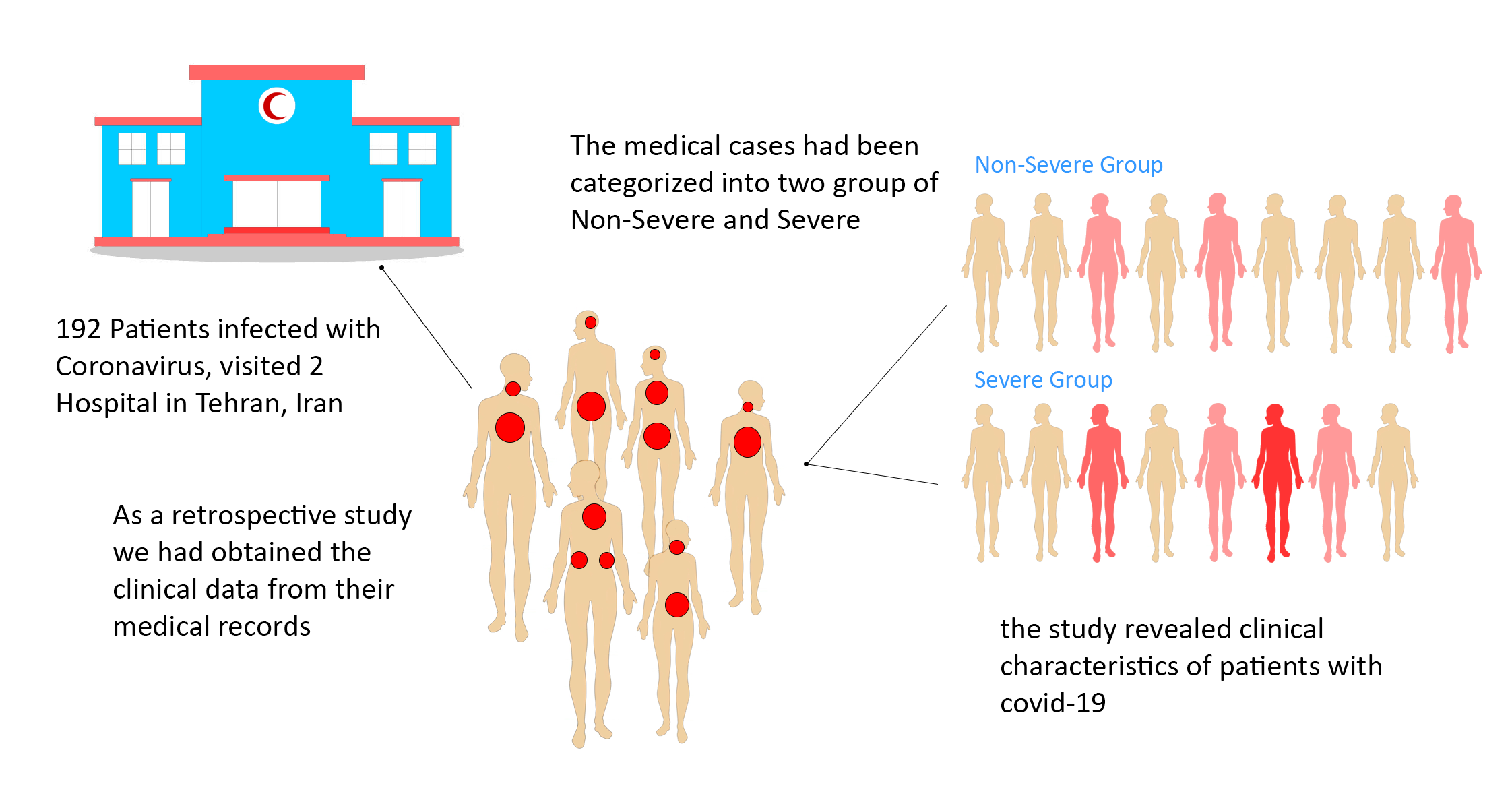

Supplement: S1 Abstract figure — (TIF) [file pone.0246314.s001.tif]
